# The Role of HSP90α in Methamphetamine/Hyperthermia-Induced Necroptosis in Rat Striatal Neurons

**DOI:** 10.3389/fphar.2021.716394

**Published:** 2021-07-19

**Authors:** Lv-shuang Liao, Shuang Lu, Wei-tao Yan, Shu-chao Wang, Li-min Guo, Yan-di Yang, Kai Huang, Xi-min Hu, Qi Zhang, Jie Yan, Kun Xiong

**Affiliations:** ^1^ Department of Anatomy and Neurobiology, School of Basic Medical Sciences, Central South University, Changsha, China; ^2^ School of Physical Education, Hunan Institute of Science and Technology, Yueyang, China; ^3^ Center for Medical Research, The Second Xiangya Hospital of Central South University, Changsha, China; ^4^ Department of Human Anatomy and Histoembryolog, School of Basic Medical Sciences, Shaoyang University, Shaoyang, China; ^5^ Department of Dermatology, Xiangya Hospital, Central South University, Changsha, China; ^6^ Department of Forensic Science, School of Basic Medical Science, Central South University, Changsha, China; ^7^ School of Basic Medical Science, Xinjiang Medical University, Urumqi, China; ^8^ Hunan Key Laboratory of Ophthalmology, Changsha, China

**Keywords:** methamphetamine, hyperthermia, heat shock protein 90 alpha, necroptosis, receptor-interacting protein 3

## Abstract

Methamphetamine (METH) is one of the most widely abused synthetic drugs in the world. The users generally present hyperthermia (HT) and psychiatric symptoms. However, the mechanisms involved in METH/HT-induced neurotoxicity remain elusive. Here, we investigated the role of heat shock protein 90 alpha (HSP90α) in METH/HT (39.5°C)-induced necroptosis in rat striatal neurons and an *in vivo* rat model. METH treatment increased core body temperature and up-regulated LDH activity and the molecular expression of canonical necroptotic factors in the striatum of rats. METH and HT can induce necroptosis in primary cultures of striatal neurons. The expression of HSP90α increased following METH/HT injuries. The specific inhibitor of HSP90α, geldanamycin (GA), and *HSP90α* shRNA attenuated the METH/HT-induced upregulation of receptor-interacting protein 3 (RIP3), phosphorylated RIP3, mixed lineage kinase domain-like protein (MLKL), and phosphorylated MLKL. The inhibition of HSP90α protected the primary cultures of striatal neurons from METH/HT-induced necroptosis. In conclusion, HSP90α plays an important role in METH/HT-induced neuronal necroptosis and the HSP90α-RIP3 pathway is a promising therapeutic target for METH/HT-induced neurotoxicity in the striatum.

## Introduction

Methamphetamine (METH) is one of the most widely abused synthetic drugs in the world. METH abuse can cause irreversible damage to many systems, such as the nervous system, the cardiovascular system, the digestive system, and the skin (Cadet et al., 2007; Paratz et al., 2016). In particular, the nervous system is one of the most important targets of METH (Degenhardt et al., 2010; Ren et al., 2016). Additionally to its strong addiction properties, METH has a strong toxic effect on the entire nervous system. Striatal neurons are extensively linked to multiple brain regions related to addiction, learning, and memory. They also play essential roles in stimulating and maintaining movement, emotional control, reward effect, and drug dependence (Alexander et al., 1986; Calabresi et al., 1997). Unfortunately, striatal neurons are very sensitive to METH-induced neurotoxicity (Granado et al., 2010; Yamamoto et al., 2010; Valian et al., 2017; Granado et al., 2018; Lu et al., 2021). Studies have shown the degeneration of dopaminergic terminals and the death of cell bodies in the striatum following METH treatment (Zhu et al., 2009; Ares-Santos et al., 2014). Apoptosis is the most focused type of neuronal cell death. However, the inhibition of the apoptotic pathway only partially inhibited METH-induced cell death (Kanthasamy et al., 2011), suggesting that other forms of cell death may also be involved in METH-induced neurotoxicity.

Necroptosis is a regulated variant of necrosis that displays a necrotic morphological feature (Font-Belmonte et al., 2020). Necroptosis can be regulated, initiated, transmitted, and executed by specific factors and blocked by several inhibitors, such as Necrostatin-1 (Nec-1) (He et al., 2009; Weinlich et al., 2017; Wang et al., 2018c; Lin et al., 2020; Hu et al., 2021; Jiang et al., 2021; Yan et al., 2021). The main factors that participate in necroptosis include receptor-interacting serine/threonine-protein 1 (RIP1), receptor-interacting serine/threonine-protein 3 (RIP3), and mixed lineage kinase domain-like protein (MLKL) (Huang et al., 2013; Ding et al., 2015; Liu et al., 2016; Shang et al., 2017; Wang et al., 2018a; Yuan et al., 2019). In this pathway, RIP3 phosphorylation is a key step in the occurrence of necroptosis. Therefore, RIP3 has been the core and characteristic molecule in the study of necroptosis (Meng et al., 2015). Although Ares-Santos’ experiments demonstrated that the neurons in the striatum showed obvious necrotic phenotypes after METH treatment (Ares-Santos et al., 2014), it is still unknown whether METH can induce necroptosis in the striatal neurons that are sensitive to METH neurotoxicity.

Hyperthermia (HT) is a critical mechanism in METH-induced neurotoxicity (Tata and Yamamoto, 2007; Krasnova and Cadet, 2009). A single medium or high dose of METH will cause HT (39–40°C), which is usually maintained for several hours (Behrouzvaziri et al., 2015; Wu et al., 2016). HT aggravates the oxidative stress and excitotoxicity caused by METH (Chauhan et al., 2014) and increases the damage to the nervous system (e.g., neuron death) (He et al., 2004). An elevated core body temperature can rapidly upregulate a variety of stress proteins, such as heat shock proteins (HSPs) (Yan et al., 2006). HSP90 belongs to one of the subfamilies of the HSPs family. It consists of two subtypes, the stress-inducible HSP90α and the constitutively expressed HSP90β (Voss et al., 2000; Sreedhar et al., 2004; Grad et al., 2010). It has been suggested that the up-regulated HSP90α acts as a molecular chaperone that stabilizes RIP1 and RIP3 and mediates necroptosis (Li et al., 2015; Li et al., 2016; Wang et al., 2018b). Our previous studies showed the significant upregulation of HSP90 mRNA in rat cortical neurons exposed to METH treatment (Xiong et al., 2017). Meanwhile, our preliminary results showed an upregulation of RIP3 and phosphorylated RIP3 (p-RIP3) in cortical brain sections of patients who died from a METH overdose (Guo et al., 2020). Based on the METH-induced HT and the possible modulatory role of HSP90α on necroptosis, we asked whether HSP90α had a significant impact on METH/HT-induced necroptosis in striatal neurons. Our investigation shed new light on the regulatory mechanism of METH/HT-induced injury.

## Materials and Methods

### Primary Striatal Neuron Cultures and *in vitro* Model Preparation

All experimental procedures were approved by the Medical Ethics Committee of the Third Xiangya Hospital of Central South University in accordance with the Guidelines for the Care and Use of Laboratory Animals (U.S. National Institutes of Health). Primary cultures of rat striatum tissues were separated from fetal Sprague-Dawley (SD) rats (embryonic day 17). In brief, rat striatum tissues were extracted with the aid of a dissecting microscope under sterile conditions. The striatum tissues were digested at 37°C for 10 min in Dulbecco’s modified Eagle’s medium (DMEM, GE Health care, Logan Utah, United States) containing 0.02% papain and then the tissues were gently triturated for 20 times and filtered through a 70 µm nylon cell sieve, followed with 5 min centrifugation. After resuspension in plating medium consisted of DMEM supplemented with 10% heat-inactivated fetal bovine serum (FBS), 5% heat-inactivated horse serum, 1 mM L-glutamine, cells were counted and plated onto flasks or plates precoated with poly-D-lysine (10 μg/ml, Sigma-Aldrich, St. Louis, United States) at a density of 6×10^5^ cells/ml. Cells were maintained at 37°C for 4 h in a 5% CO_2_ incubator after plating, followed by replacing the plating medium with neurobasal medium (Thermo Scientific, MA, United States) supplemented with 2% B27 (Thermo Scientific). Half of the culture media were replaced every 2 days. On the 7th day, the cultures were exposed to indicated concentration of METH applied by Changsha City Public Security Bureau, China, cultured in a 5% CO_2_ incubator at 39.5°C for 3 or 6 h. The cell cultures in the normal control group were still cultured in a 5% CO_2_ incubator at 37°C in parallel.

### *In vivo* METH Administration

Male SD rats, each weighing 200–210 g at the beginning of the experiment, were obtained from the Animal Center of Central South University. Animals were housed in a temperature (23 ± 2°C) and humidity (50 ± 5%) controlled animal facility. All experimental rats were housed together in 50 × 35 × 20 cm cages (*n* = 3/cage) and were maintained on a 12 h light/dark cycle with free access to food and water. METH (10 mg/kg) or saline were administered to rats every 2 h in four successive intraperitoneal (i.p.) injections. Rats were sacrificed by decapitation at 1, 12, or 24 h after the last injection of METH or saline. The rectal temperature of rats was monitored by an electronic thermometer throughout METH treatment, at 30 min after each injection, and rectal temperature 30 min before METH treatment was considered as the baseline.

### Drug Preparation and Administration

For *in vitro* experiments, we pretreated primary cultured neurons with 20 µM Nec-1 (Sigma-Aldrich) diluted with DMSO for 2 h before conducting METH and 39.5°C treatment to determine the rate of necroptosis (Vieira et al., 2014). 50, 100, 300, and 900 nM HSP90 inhibitor Geldanamycin (GA) (Cell Signaling Technology, MA, United States) diluted with DMSO were picked and added into the primary striatal neuronal medium for 24 h before conducting METH and 39.5°C treatment. For *in vivo* experiments, GA was diluted with 1% DMSO (diluted with saline) to a final concentration of 1.6 mΜ. After anesthetized by i.p. injection of 1% pentobarbital sodium (6 ml/kg), rats were placed into a stereotaxic frame. A 23-gauge stainless steel guide cannula attached to a 10 μl Hamilton® syringe was stereotactically inserted (coordinates: striatum: AP + 0.9 mm, lateral −2.2 mm, −4.4 mm beneath the pial surface). 5 μl GA or dilute DMSO was injected 1 h before METH administration at a rate of 0.5 μl/min (Wen et al., 2008; Yin et al., 2017). Following the intracerebral injection, rats were removed from the stereotaxic device and maintained at a rectal temperature of 37°C throughout surgery and recovery.

### Lentivirus Infection in Primary Cultured Striatal Neurons

The lentivirus kit containing three shRNA sequences of HSP90α gene and one negative control sequence was purchased from Jikai gene (Shanghai, China). The sequences are as following: Hsp90aa1-RNAi Sequence 1: GAC​AGC​AAA​CAT​GGA​GAG​AAT, Hsp90aa1-RNAi Sequence 2: GCT​TTC​AGA​GCT​GTT​GAG​ATA, Hsp90aa1-RNAi Sequence 3: AAG​TAC​ATT​GAT​CAA​GAA​GAA. Firstly, we conducted the pretest study to explore the suitable concentration of the lentivirus for infecting primary cultured striatal neurons. Four concentrations (MOI:1, MOI:3, MOI:5, and MOI:10) of the negative control lentivirus were applied to infect primary cultured neurons on the 4th day after planting in plates. Then change the medium after infecting for 24 h, and continue to infect for 72 h. The GFP positive cells were captured by a fluorescence microscope. The formal experiment was carried out for infecting primary cultured neurons at MOI:3 after infecting for 72 h. The rate of knocking down of HSP90α was detected by western blot.

### Propidium Iodide Staining

Propidium iodide (PI) staining was used to identify necrotic cells (Shang et al., 2014; Guo et al., 2020). At the indicated time points, cell cultures on the coverslips were washed three times with PBS and then incubated with 10 μg/ml PI dye in a 5% CO_2_ incubator at 37°C for 10 min. Then, cell cultures were perfused with 4% paraformaldehyde (PF) for 20 min at room temperature (RT) followed by washing three times in PBS buﬀer and covered the slides with an anti-fading mounting solution containing DAPI (Vector Laboratories, CA, United States). Images acquired with a fluorescence microscope using the same exposure time were captured for five random fields of each group. The percentages of PI-positive cells, which were analyzed in every intact captured image using ImageJ software (National Institutes of Health, MD, United States), are calculated from the number of PI-positive cells divided by the number of DAPI-positive cells.

### Lactate Dehydrogenase Release Assay

The release of lactate dehydrogenase (LDH) into the extracellular space/supernatant is considered to be an important feature of broken cell membrane integrity (Kumar et al., 2018; Parhamifar et al., 2019). The LDH assay is a non-radioactive colorimetric assay. For *in vitro* analysis, we used the LDH cytotoxicity assay kit (Beyotime, Shanghai, China) to determine the LDH released from necrotic cells in each group. In brief, cell culture plates were centrifuged at 1,500 rpm for 5 min, followed by harvesting the cell-free culture supernatants from each well of the plate and then incubated with the working reagent mixture at RT for 30 min. Subsequently, the optical density of each well in the assay was measured with a microplate reader at the wavelength of 490 and 650 nm. The optical density is directly proportional to the LDH activity and the percentage of necrotic cells. The percentages of necrotic cell death are equal to the optical density of the treated group minus control group/LDH releasing reagent treated group minus control group, which was calculated from three independent experiments. The LDH cytotoxicity assay kit (Nanjing Jiancheng Bioengineering Institute, Nanjing, China) was used for the *in vivo* analysis according to the manufacturer’s instructions. In brief, rats were anesthetized with 10% chloral hydrate and then sacrificed by decapitation. Rat striatum tissues were quickly removed and immediately homogenized in 0.86% ice-cold NaCl by sonication, then tissue homogenates were centrifuged at 2,500 rpm for 10 min. The supernatant solutions were collected before incubation with the working reagent mixture for 30 min at 37°C. The optical density of each group was detected with a microplate reader at the wavelength of 450 nm. The percentage of necrotic cell death was measured by the color intensity of treated group minus negative control group/standard group minus blank control group, according to the manufacturer’s instructions.

### Immunofluorescence Staining

For *in vitro* experiments, at the indicated time points, cell cultures on the coverslips were washed three times with PBS and fixed with 4% PF for 20 min. For *in vivo* experiments, the rats received intracardiac perfusion with saline and 4% PF. The brains were then dehydrated in a series of 15 and 30% sucrose solutions before dissection. Coronal slices (20 μm thickness) encompassing the striatum were collected and then used for staining. After three times washed in PBS again, cell cultures on the coverslips and slices were blocked at RT in blocking buﬀer, i.e., PBS containing 0.3% Triton X-100 and 5% normal bovine serum for 1–2 h. Incubate cell coverslips and slices with primary antibodies against the following targets at 4°C overnight: HSP90α (1:100, Abcam, Cambridge, United Kingdom), RIP3 (1:100, Sigma-Aldrich), TH (1:200, Abclonal, Wuhan, China; 1:200, Santa Cruz, TX, United States), Map-2 (1:100, Proteintech Group, IL, United States). The next day, coverslips and slices were moved to RT for 30 min, washed three times with PBS, and then incubated with Alexa-conjugated secondary antibodies (1:200, Jackson ImmunoResearch, PA, United States) for 2 h at RT with gentle fluctuation. The coverslips were washed three times with PBS, followed by covering with an anti-fading mounting solution containing DAPI (Vector Laboratories). All the staining procedures were in parallel and images were captured using the same settings at five random fields of view on each coverslip with a fluorescence microscope.

### Western Blot Detection

At the indicated time points, the cultured neurons and the rat striatum tissues were harvested, washed twice with ice-cold PBS, and then dissociated with RIPA buﬀer contained 1% phosphorylated inhibitors and 1% protease inhibitors (CWBIO, Beijing, China). The extracts were centrifuged at 12,000 rpm for 20 min at 4°C, and the supernatant was transferred to a new tube. We measured the protein concentration of these samples by BCA assay. After unifying the concentration, we added 5× loading buffer, boiled them for 5 min, centrifuged them at 1,000 rpm for 5 min. The total loading protein for each lane is 20 µg. The samples were loaded in 8–12% SDS-PAGE gel and then transferred the protein from the gel to a PVDF membrane (Millipore, MA, United States) in ice-cold transfer buffer. After washing with TBST for once, the membranes were blocked with 5% skim milk at RT for 1–2 h to wipe off the non-specific protein band. The membranes were incubated with primary antibody (RIP3, 1:1,000; HSP90α, 1:1,000; MLKL, 1:500; GAPDH, 1:5,000 (Beyotime) at 4°C overnight. The next day, after washing the membrane with TBST three times, the membranes were incubated with the homologous HRP-conjugated secondary antibody (1:2,500, Jackson ImmunoResearch) at RT for 1.5 h. And then high sensitivity chemiluminescence reagent (CWBIO) was used to visualize the immunoreactive bands. Integrated density values of specific proteins, which quantified by ImageJ software, were normalized to the GAPDH values.

### Phos-Tag™ SDS-PAGE

The concentration of acrylamide SDS-PAGE gel for RIP3 and MLKL was 8%, the concentration of phos-tag™ (Wako Pure Chemical Industries, Japan) was 50 µM. The protocol was similar to western blot (WB) but the operation before transferring onto the PVDF membrane. Before transferring onto the membrane, the gel needed to be washed with transferring buffer containing 1 mM EDTA for 15 min, then with transferring buffer without EDTA for 15 min to get rid of Mn^2+^.

### Co-Immunoprecipitation

Primary cultured neurons were harvested and lysed in cold immunoprecipitation (IP) extraction buffer containing 1% phosphatase and 1% protease inhibitor and the protein solution medium was separated by centrifugation at 12,000 rpm at 4°C for 20 min. Four micrograms of HSP90α antibody and IgG (Abclonal) were pre-incubated with protein A/G agarose beads (Santa Cruz) for 8 h at RT and washed with GLB^+^ buﬀer for five times. Then 500 µg protein from extracted protein was incubated with protein A/G agarose beads coupled with primary antibody at 4°C for 24 h with gentle fluctuating. On the following day, the mixture was pre-washed five times with cold GLB^+^ buffer, and proteins were eluted with prepared 1× loading buffer by boiling for 5 min and centrifuge for 5 min at 10,000 rpm to collect the supernatant and subjected to SDS-PAGE.

### Statistical Analysis

To ensure consistency of the results, all experiments were replicated at least three times. Figure panels were assembled using Photoshop CC (Adobe Systems Incorporated, CA, United States). The measurement data are analysed by GraphPad Prism 5 (GraphPad Software Inc., CA, United States) and presented as the mean ± standard deviation. Statistical significance was set at *p* < 0.05.

## Results

### METH Treatment Increased the Core Body Temperature and Up-Regulated LDH Activity and the Molecular Expression of Canonical Necroptotic Factors in the Striatum of Rats

**FIGURE 1:**
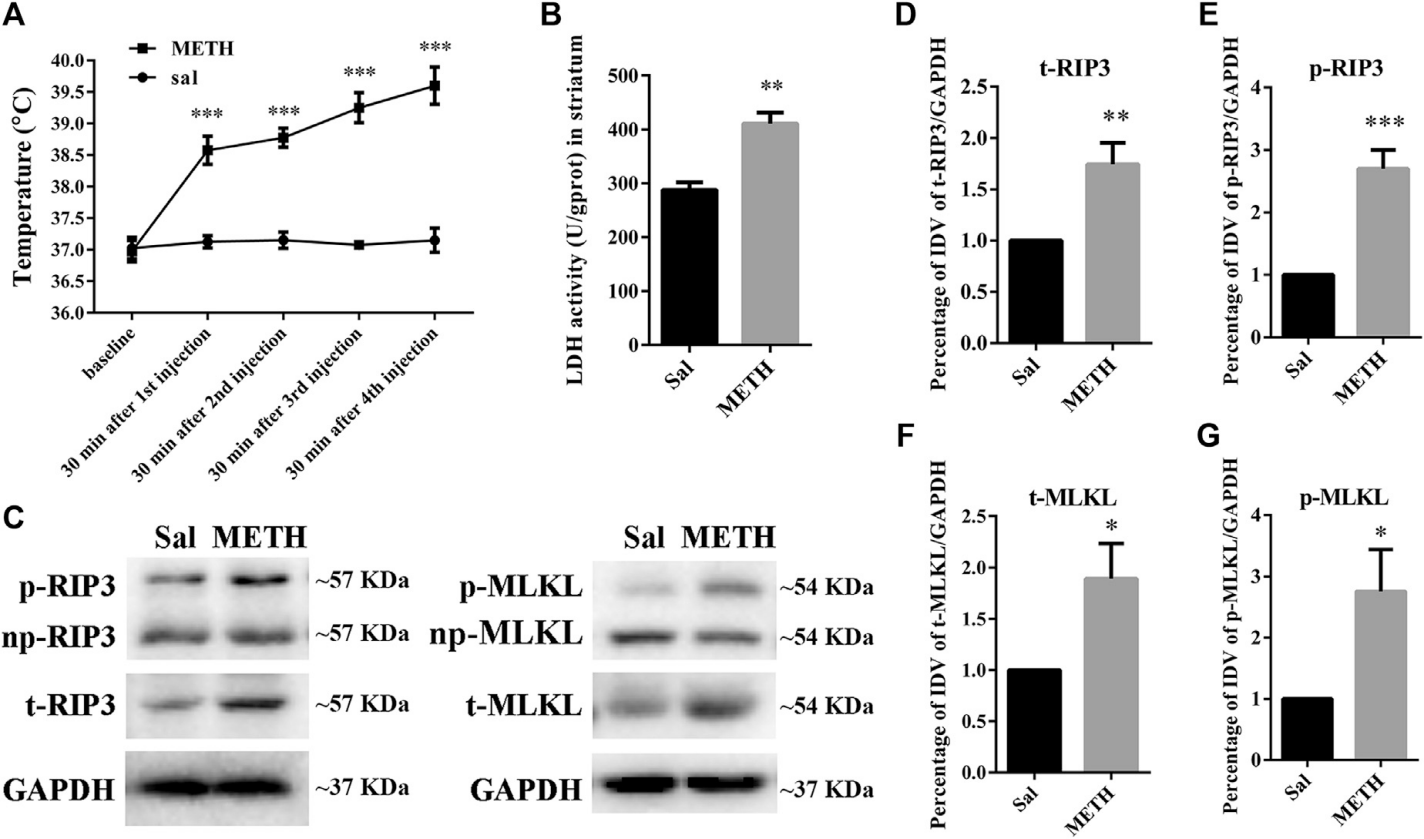
Changes in core body temperature, LDH activity, and canonical necroptotic factors expression in the striatum of rats following METH administration. Rats were sacrificed by decapitation at 24 h after the last injection of METH (4 × 10 mg/kg, every 2 h, i.p.) or saline. **(A)** METH treatment increased core body temperature. Data were analyzed by RM two-way analysis of variance (ANOVA) which was carried out with GraphPad Prism 5 software (*n* = 3). ***p < 0.001 vs. baseline. **(B)** Necrosis in the striatum of rats was determined by LDH cytotoxicity assay. Data were analyzed by unpaired 2-tailed Student’s t test (*n* = 3). **p < 0.01 vs. Sal group. **(C)** p-RIP3, RIP3, p-MLKL, and MLKL protein levels in the rat striatum were detected by phos-tag SDS-PAGE and WB after METH administration. **(D–G)** Statistical analysis of WB of p-RIP3, RIP3, p-MLKL, and MLKL expression. np, non-phosphorylated protein; t, total protein; Sal, saline group. Data were analyzed by unpaired 2-tailed Student’s t test (*n* = 3). *p < 0.05, **p < 0.01, ***p < 0.001 vs*.* Sal group.

HT is an important contributor to METH-induced neurotoxicity (Yamamoto et al., 2010). It usually reaches a peak 30 min after METH treatment. To determine whether our METH and HT insult rat model was successful, we measured core body temperatures before the first METH or saline injection and 30 min after each METH or saline injection. As shown in Figure 1A, there was no significant difference in the basal body temperature of rats treated with a saline solution. Compared to the saline controls, METH treatment (4 × 10 mg/kg, every 2 h, i.p.) significantly increased core body temperatures. The temperatures also increased with the number of injections and reached about 39.5°C 30 min after the fourth METH injection, similar to the previous study (Chauhan et al., 2014). These results indicate that the rats treated with METH displayed higher temperatures than the rats treated with saline. We also conducted LDH cytotoxicity assays *in vivo* (Figure 1B). Compared with the saline group, we observed a higher LDH release in the METH treatment group.

The increased expression of RIP3 and MLKL mRNA or protein *in vivo* has been reported in various diseases or physiological conditions (Guo et al., 2020). The activated forms of RIP3 and MLKL are optimal biomarkers to detect necrosis and to assess the diagnosis or prognostic of diseases related to necrotic injuries (He et al., 2016; Hu et al., 2021). Therefore, we first speculated whether METH administration could cause the corresponding molecular changes in the striatum of rat brains (Figures 1C–G). The phos-tag SDS-PAGE results (the upper bands) showed that the expression of p-RIP3, total RIP3 (t-RIP3), and phosphorylated MLKL (p-MLKL) in the rat striatum were higher in the binge METH treatment group than in the control groups. Together, these results indicate that METH administration and METH-induced HT may induce necroptosis in the striatum of rats.

### METH and HT Induced Necroptosis in Primary Cultures of Striatal Neurons

**FIGURE 2:**
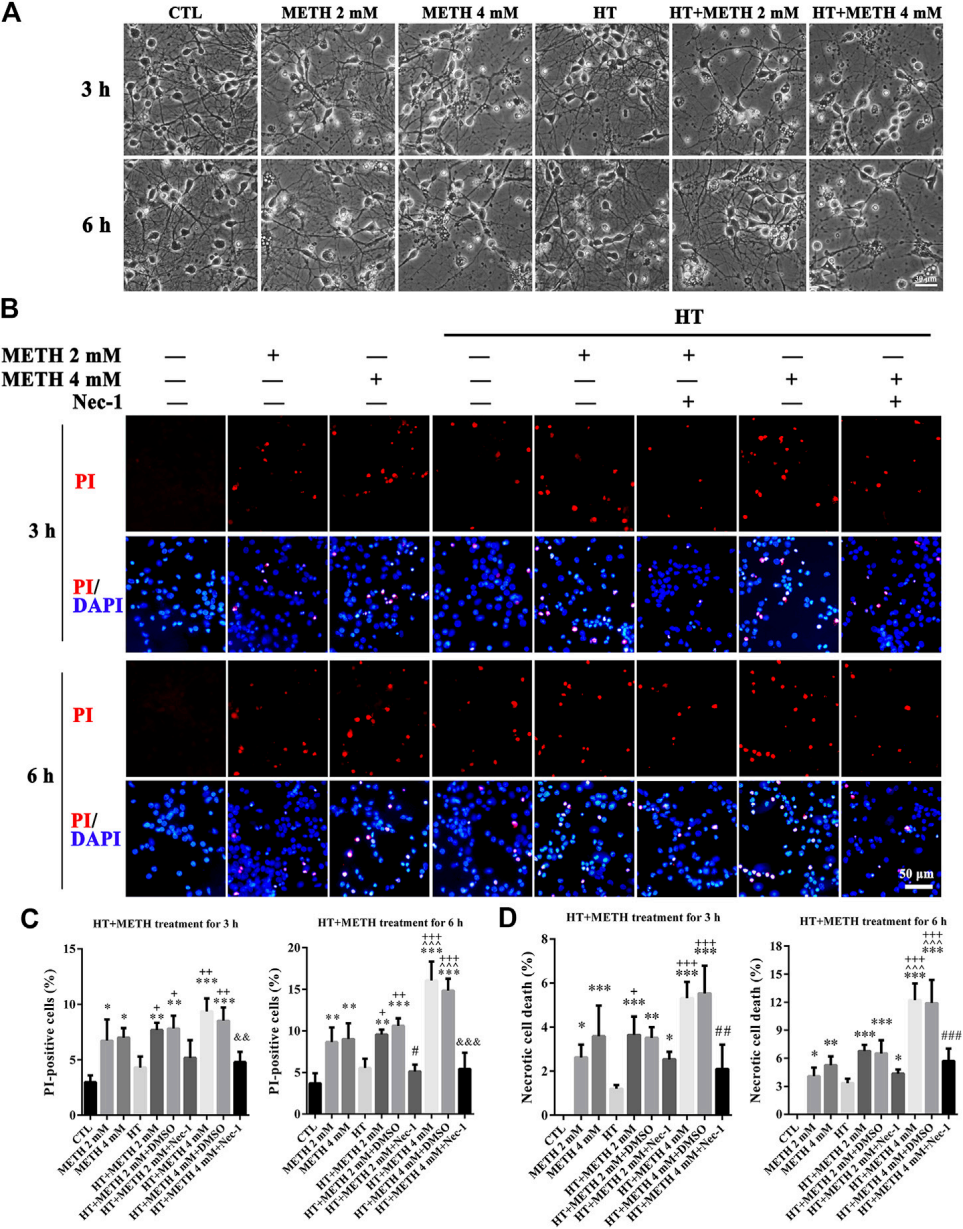
METH/HT-induced cell morphological changes and Nec-1 pre-treatment attenuated the METH/HT-induced necrosis in striatal neurons. **(A)** Cell morphological changes of striatal neurons treated with METH for 3 and 6 h under a light microscope. *Scale bar* = 30 μm **(B)** PI (red)/DAPI (blue) double staining of striatal neurons pre-treated with Nec-1 after 3 and 6 h of METH/HT. *Scale bar* = 50 μm. **(C)** Statistical analysis of the PI/DAPI double staining of necrotic cells. Data were analyzed by one-way ANOVA, followed by a Tukey multiple comparisons posttest (*n* = 3). *p < 0.05, **p < 0.01, ***p < 0.001 vs. CTL group;  ^ ^ ^*p* < 0.05 vs. METH 4 mM; +*p* < 0.05, ++*p* < 0.01, +++*p* < 0.001 vs. HT; #*p* < 0.05 vs. HT + METH 2 mM group; &&*p* < 0.01, &&&*p* < 0.001 vs. HT + METH 4 mM group. **(D)** The percentage of necrotic neuron death after METH/HT treatment and pre-treatment with Nec-1 was determined with LDH release assays. Data were analyzed by one-way ANOVA, followed by a Tukey multiple comparisons posttest (*n* = 3). *p < 0.05, **p < 0.01, ***p < 0.001 *vs.* CTL group;  ^ ^ ^*p* < 0.05 vs. METH 4 mM; +*p* < 0.05, +++*p* < 0.001 vs. HT; ##*p* < 0.01, ###*p* < 0.001 vs. HT + METH 4 mM group.

The purity of the striatal neuronal cells was assessed on the 7th day by immunoreactivity to microtubule association protein-2 (Map-2). In all, 90.5 ± 2.06% of the cells were Map-2 positive (Supplementary Figure 1). To observe whether striatal neurons are injured by METH and HT, we incubated our cultured neurons with 2 and 4 mM METH at 39.5°C for 3 or 6 h and observed the change in neuronal morphology under a light microscope. The results showed that 2 mM of METH + 39.5°C for 3 h damaged the neurites. The degree of damage to the neurites increased with a higher concentration of METH and HT duration time. After exposure to 4 mM of METH + 39.5°C for 6 h, the neurons were severely damaged and presented obvious necrosis-like features as the neuronal body began to swell and the massive neurites broke and fractured (Figure 2A). These results showed that METH and HT induced neuronal necrosis-like cell death.

To determine whether necroptosis occurred in primary cultures of striatum neurons exposed to METH and HT, we employed the necroptosis inhibitor Nec-1 and two necrosis detecting methods. We did not observe any apparent PI-positive cells (necrotic cells) after PI staining in the control group. We detected necrotic cells after treating the neurons with METH for 3 and 6 h and HT exacerbated the injury. The number of necrotic cells increased with METH concentration and HT treatment time (Figure 2B). The quantitative analysis of the number of necrotic cells showed that their number increased dramatically in the METH and HT treatment groups. However, the number of necrotic cells in the Nec-1 + 4 mM of METH + 39.5°C treatment group was lower than in the 4 mM of METH + 39.5°C group after 6 h (Figure 2C). The LDH release results also showed that the treatment with Nec-1 significantly decreased the number of necrotic cells induced by METH and HT for 6 h. The multiple comparisons test among groups showed that necrotic cell death in HT + METH 4 mM treatment for 6 h group was more than single HT and single METH treatment for 6 h group (Figure 2D). Thus, we performed the analysis 6 h after treatment to further study the mechanism of METH/HT-induced neuronal injury. Collectively, these results suggest that METH and HT induce necroptosis in primary cultures of striatum neurons.

**FIGURE 3:**
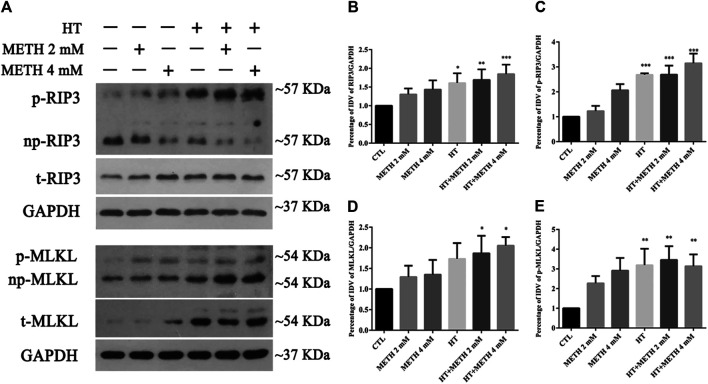
Detection of RIP3, p-RIP3, MLKL, and p-MLKL expression by WB after METH/HT injury in striatal neurons. **(A)** p-RIP3, RIP3, p-MLKL, and MLKL protein levels in primary cultures of striatal neurons detected by phos-tag SDS-PAGE and WB after 6 h of METH treatment at 39.5°C. **(B–E)** Statistical analysis of WB of p-RIP3, RIP3, p-MLKL, and MLKL expression. Data were analyzed by one-way ANOVA, followed by a Tukey multiple comparisons posttest (*n* = 3). *p < 0.05, **p < 0.01, ***p < 0.001 vs. CTL group.

We detected the changes in the expression of several canonical necroptotic molecules following METH and HT treatment (Figure 3A). Our results showed that the level of p-RIP3, t-RIP3, and p-MLKL was higher in both 39.5°C treatment groups than in the control groups. The band thickness of p-RIP3, t-RIP3, p-MLKL, and t-MLKL was remarkably increased in the 4 mM METH + 39.5°C group. The quantitative analysis of the WB showed that METH slightly increased p-RIP3, t-RIP3, p-MLKL, and t-MLKL levels. However, the co-treatment with METH and HT significantly increased the expression of both canonical necroptotic molecules. The level of p-RIP3, t-RIP3, *p*-MLKL, and t-MLKL increased rapidly in the cells treated with 4 mM METH at 39.5°C for 6 h (Figures 3B–E). Thus, we performed further experiments at the concentration of 4 mM of METH.

### HSP90α Was Involved in METH/HT-Induced Necrosis in Primary Cultures of Striatal Neurons

**FIGURE 4:**
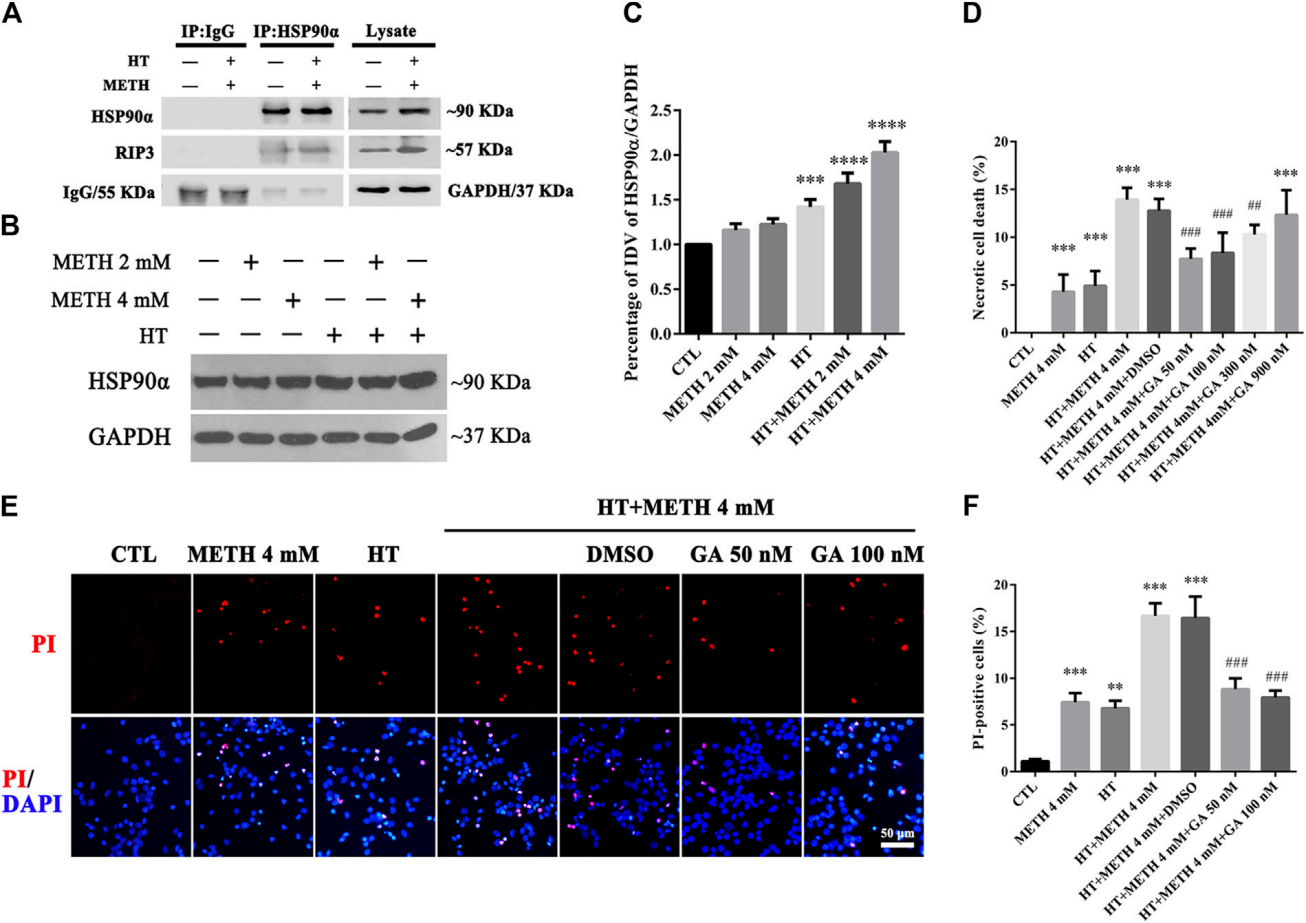
HSP90α interacts with RIP3 and GA pretreatment protected primary striatal neurons from METH/HT-induced necrosis. **(A)** Detection of HSP90α and RIP3 interaction in primary cultures of neurons by IP **(B)** WB of HSP90α expression after METH treatment for 6 h at 39.5°C. **(C)** Statistical analysis of WB of HSP90α expression. Data were analyzed by one-way ANOVA, followed by a Tukey multiple comparisons posttest (*n* = 3). ***p < 0.001; ****p < 0.0001 vs. CTL group **(D)** Percentage of necrotic neuron death after METH/HT treatment and pre-treatment with GA as determined by LDH release assays. Data were analyzed by one-way ANOVA, followed by a Tukey multiple comparisons posttest (*n* = 3). ***p < 0.001 vs. CTL group; ##*p* < 0.01, ###*p* < 0.001 vs. HT + METH 4 mM group **(E)** PI (red)/DAPI (blue) double staining of striatal neurons after METH/HT and pre-treatment with GA. *Scale bar* = 50 μm. **(F)** Statistical analysis of PI/DAPI double staining of necrotic cells. Data were analyzed by one-way ANOVA, followed by a Tukey multiple comparisons posttest (*n* = 3). **p < 0.01, ***p < 0.001 vs. CTL group; ###*p* < 0.001 vs. HT + METH 4 mM group.

HSP90α acts as a molecular chaperone to stabilize RIP3 and mediate necroptosis (Li et al., 2016). Our IP results showed that the interaction between HSP90α and RIP3 was increased in the 4 mM METH + 39.5°C treatment for 6 h group compared to that in the control group (Figure 4A). Our WB results showed that a single METH treatment did not upregulate the expression of HSP90α. HSP90α expression was, however, significantly increased following METH and HT co-treatment (Figure 4B). The quantitative analysis of the WB showed that HSP90α expression significantly increased in cells treated with 4 mM METH at 39.5°C for 6 h (Figure 4C). These results suggest that the increase in HSP90α levels may be related to METH/HT-induced necroptosis in primary cultures of striatal neurons.

We found above that the expression of HSP90α increased following METH and HT treatment and detected a potential interactive relationship between RIP3 and HSP90α. Therefore, we predicted that HSP90α might be involved in METH/HT-induced necrosis in primary cultures of striatal neurons. The cells were treated with GA, an HSP90α inhibitor, for 24 h before adding METH and HT to determine whether HSP90α could mediate striatum neuronal necroptosis. Firstly, we found the best working concentration of GA with literature reviews and experimental verifications (Chen et al., 2012). The LDH release results showed that a pre-treatment with 50 nM, 100 nM, and 300 nM GA protected the striatum neurons from necrosis following METH and HT insults (Figure 4D). The PI staining also indicated that treatment with 50 and 100 nM GA effectively reduced the number of PI-positive cells after METH and HT insults (Figure 4E). The statistical analysis for the PI staining showed that the number of necrotic cells was remarkably reduced in all the GA pre-treatment groups as compared to that of the METH/HT groups (Figure 4F). These results suggest that GA could, at least partially, rescue METH/HT-induced necrosis in primary cultures of striatal neurons.

**FIGURE 5:**
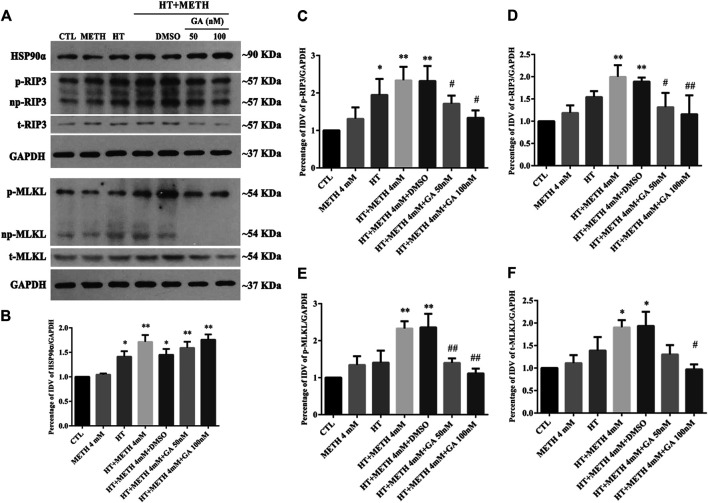
Detection of HSP90α, RIP3, p-RIP3, MLKL, and *p*-MLKL in striatal neurons following METH/HT injury and pre-treatment with GA. **(A)** WB of HSP90α, RIP3, p-RIP3, MLKL, and p-MLKL after treatment with METH/HT for 6 h and pre-treatment with 50 and 100 nM GA. **(B–F)** Statistical analysis of WB of HSP90α, RIP3, p-RIP3, MLKL, and p-MLKL expression. Data were analyzed by one-way ANOVA, followed by a Tukey multiple comparisons posttest (*n* = 3). *p < 0.05, **p < 0.01 vs. CTL group; #*p* < 0.05, ##*p* < 0.01 vs. HT + METH 4 mM group.

As HSP90α might decrease METH/HT-induced necroptosis in striatal neurons, we next investigated how HSP90α might regulate the process of protection. First, we measured the level of RIP3 (acting as a client and downstream molecule of HSP90α), p-RIP3, and its downstream molecules MLKL and p-MLKL following treatment with GA in the METH/HT groups. The phos-tag SDS-PAGE results showed that METH and HT increased the level of t-RIP3, p-RIP3, t-MLKL, and p-MLKL. This effect was reversed in cells pre-treated with GA (Figure 5A). The statistical analysis showed that GA treatment decreased the expression level of t-RIP3, p-RIP3, t-MLKL, and p-MLKL (Figures 5B–F). GA pre-treatments did not affect the expression of HSP90α following METH and HT. This may be because GA mainly binds to the N-terminal ATP-binding domain of HSP90 and inhibits its ATP-dependent chaperone activity (Guo et al., 2005; Hermane et al., 2019). These results suggest that GA can protect striatal neurons from METH/HT-induced necroptosis by decreasing the levels of t-RIP3/MLKL and p-RIP3/MLKL. Collectively, the above results indicate that HSP90α might be involved in METH/HT-induced necrosis in primary cultures of striatal neurons.

### HSP90α shRNA Partially Protected Primary Cultures of Striatal Neurons From METH/HT-Induced Necroptosis

**FIGURE 6:**
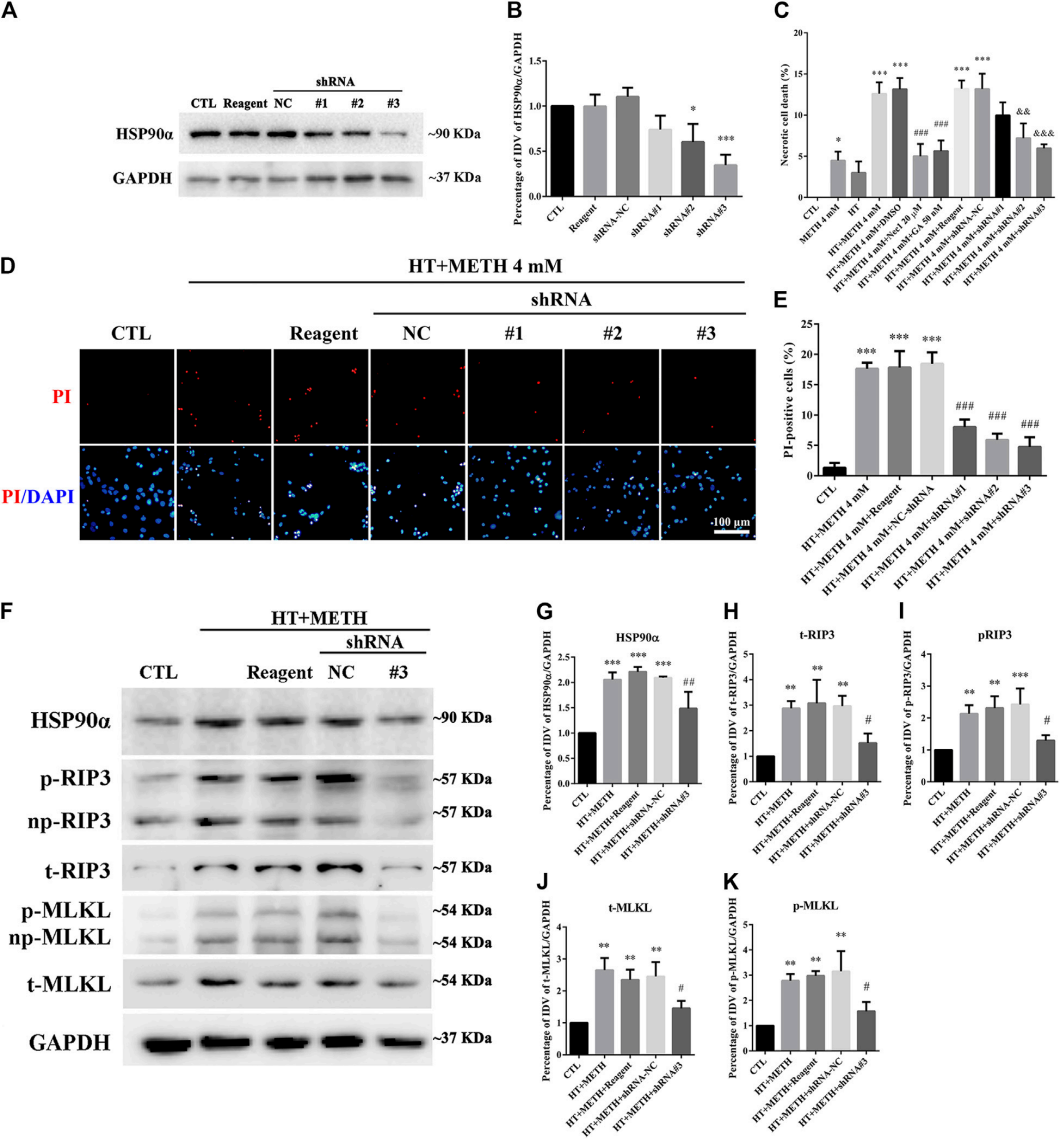
*HSP90α* shRNA protected primary striatal neurons from METH/HT-induced necrosis. **(A)** The protein level of HSP90α after infection with lentivirus in primary cultures of striatal neurons detected by WB. Reagent, infection reagent without shRNA group; NC, infection reagent with negative control of shRNA group; #1-3, infection reagent with the indicated sequence of *HSP90α* shRNA group **(B)** Statistical analysis of HSP90α WB. Data were analyzed by one-way ANOVA, followed by a Tukey multiple comparisons posttest (*n* = 3). *p < 0.05, ***p < 0.001 vs. CTL group. **(C)** The percentage of necrotic neuron death after METH/HT treatment and pre-treatment with Nec-1, GA, and *HSP90α* shRNA was determined with LDH release assays. Data were analyzed by one-way ANOVA, followed by a Tukey multiple comparisons posttest (*n* = 3). *p < 0.05, ***p < 0.001 vs. CTL group; ###*p* < 0.001, &&*p* < 0.01, &&&*p* < 0.001 vs. HT + METH 4 mM group, respectively. **(D)** PI (red)/DAPI (blue) double staining of striatal neurons after METH/HT treatment and pre-treatment with *HSP90α* shRNA. *Scale bar* = 50 μm. **(E)** Statistical analysis of the PI/DAPI double staining of necrotic cells. Data were analyzed by one-way ANOVA, followed by a Tukey multiple comparisons posttest (*n* = 3). ***p < 0.001 vs. CTL group; ###*p* < 0.001 vs. HT + METH 4 mM group. (**F)** WB of HSP90α, RIP3, p-RIP3, MLKL, and p-MLKL after METH/HT treatment for 6 h and pre-treatment with HSP90α shRNA. **(G–K)** Statistical analysis of WB of HSP90α, RIP3, p-RIP3, MLKL, and p-MLKL expression. Data were analyzed by one-way ANOVA, followed by a Tukey multiple comparisons posttest (*n* = 3). **p < 0.01, ***p < 0.001 vs. CTL group; #*p* < 0.05, ##*p* < 0.01 vs. HT + METH 4 mM group.

Four lentivirus concentrations (MOI:1, MOI:3, MOI:5, MOI:10) were used to explore the suitable lentivirus infective concentration for further experiments. The results showed that the GFP expression at MOI:3 and MOI:5 was optimal with a GFP-positive cell rate above 80% (Supplementary Figure 2). Thus, MOI: 3 was selected for further experiments. The results from the WB revealed that shRNA #2-3 lentiviruses pretreatment reduced the expression of HSP90α. The shRNA #3 sequence had the highest silencing efficiency (Figures 6A,B). The LDH release results confirmed that necrosis was significantly decreased in the shRNA #2-3 lentiviruses + METH and HT group as compared to the METH and HT group without lentiviral treatment (Figure 6C). We observed few PI-positive cells (necrotic cells) in the control group and an increased number of necrotic cells following METH and HT treatment for 6 h. The number of necrotic cells was reduced in the shRNA #1-3 lentiviruses + METH and HT group compared to that in the METH and HT group without lentiviral treatment (Figure 6D). The quantitative analysis of the necrotic cell numbers showed that it was lower in the shRNA #1-3 lentiviruses + METH and HT group than in the METH and HT group. Of the three sequences, the shRNA #3 lentivirus-transfected group had the lowest number of necrotic cells (Figure 6E). Therefore, the shRNA #3 lentivirus sequence was chosen in the following infective experiment. Collectively, these results suggest that *HSP90α* shRNA decreased METH/HT-induced necrosis in striatal neurons.

**FIGURE 7:**
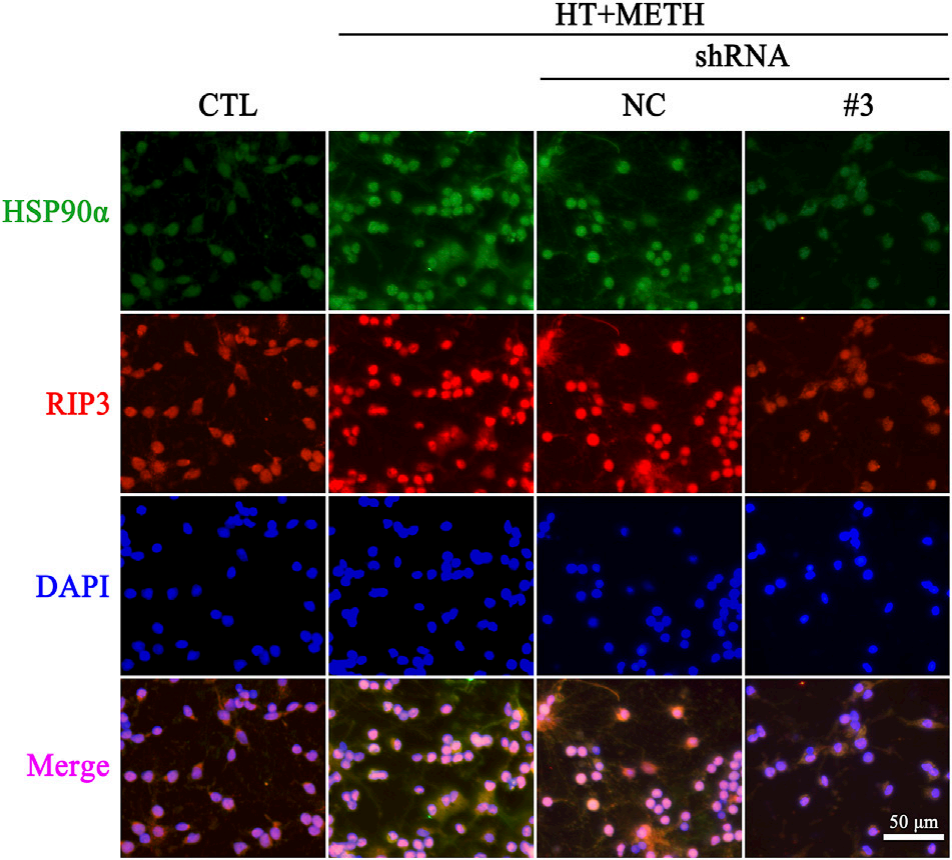
IF of HSP90α (green) and RIP3 (red) in striatal neurons following METH/HT injury and pre-treatment with *HSP90α* shRNA. *Scale bar* = 50 μm.

To further investigate the regulatory role of HSP90α in METH/HT-induced necrosis, we inhibited the function and expression of HSP90α using a specific shRNA. The phos-tag SDS-PAGE results showed that the upregulation of HSP90α, t-RIP3, p-RIP3, t-MLKL, and *p*-MLKL induced by METH/HT decreased in the shRNA #3 lentivirus-transfected group compared with that in the METH/HT-treated group (Figure 6F). Our statistical analysis showed that the expression level of HSP90α, t-RIP3, p-RIP3, t-MLKL, and p-MLKL increased in the HT + METH groups, the HT + METH + Reagent group, and the HT + METH + NC group but decreased in the HT + METH + #3 *HSP90α* shRNA group (Figures 6G–K). The immunofluorescence (IF) staining showed that both the expression of HSP90α (green) and RIP3 (red) increased following METH and HT treatment. However, the knockdown of HSP90α by shRNA not only attenuated the IF intensity of HSP90α but also of RIP3 (Figure 7). Taken together, these results suggest that HSP90α inhibition can partially protect striatal neurons from METH/HT-induced necroptosis by decreasing the expression of RIP3 and MLKL.

### Inhibition of HSP90α Protected Striatal Neurons from METH/HT-Induced Necroptosis *in vivo*

**FIGURE 8:**
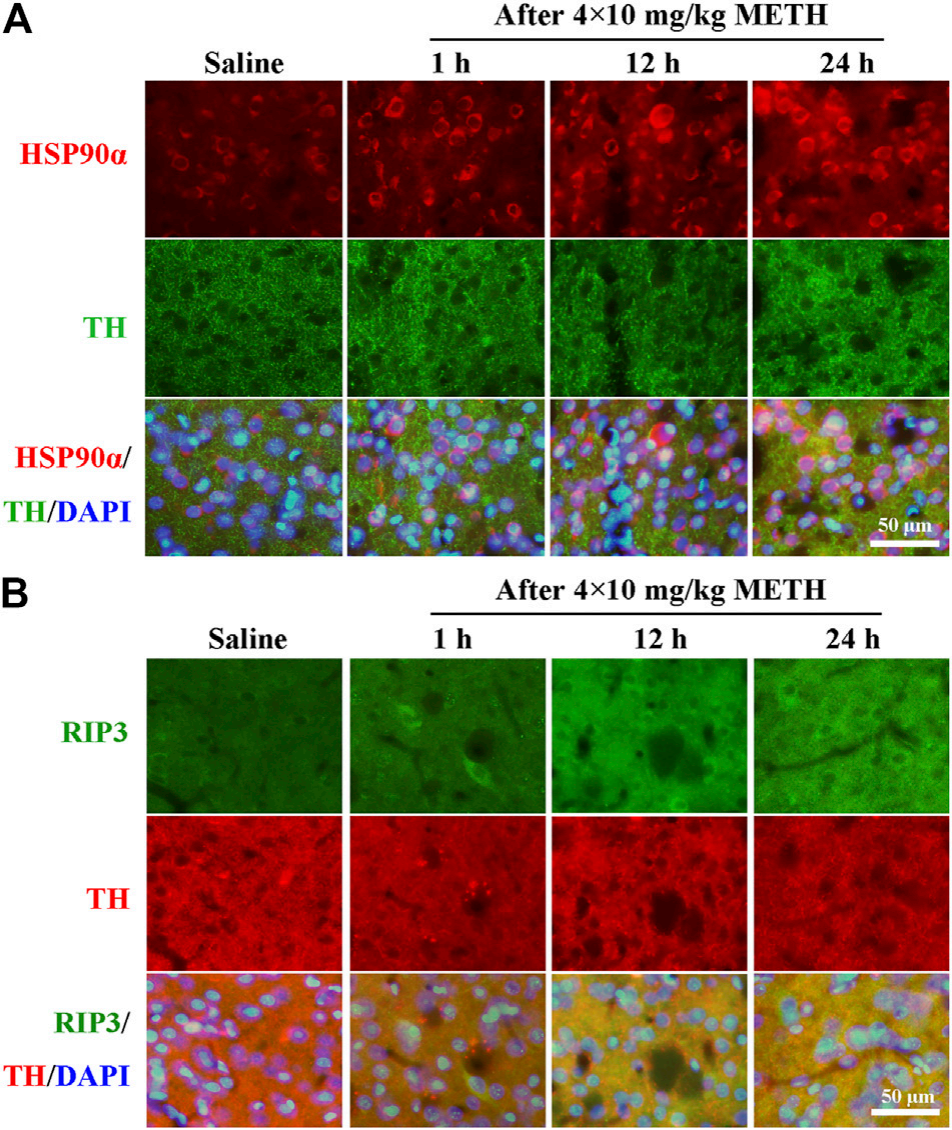
Detection of HSP90α and RIP3-positive cells in the rats’ striatum following METH administration by IF. **(A)** IF staining of HSP90α (red) and tyrosine hydroxylase (TH) (green) 1, 12, and 24 h after the last injection of a binge dose of METH (4 × 10 mg/kg, every 2 h, i.p.) or saline. **(B)** IF staining of RIP3 (green) and TH (red) 1, 12, and 24 h after the last injection of a binge dose of METH. *Scale bar* = 50 μm in all panels.

**FIGURE 9:**
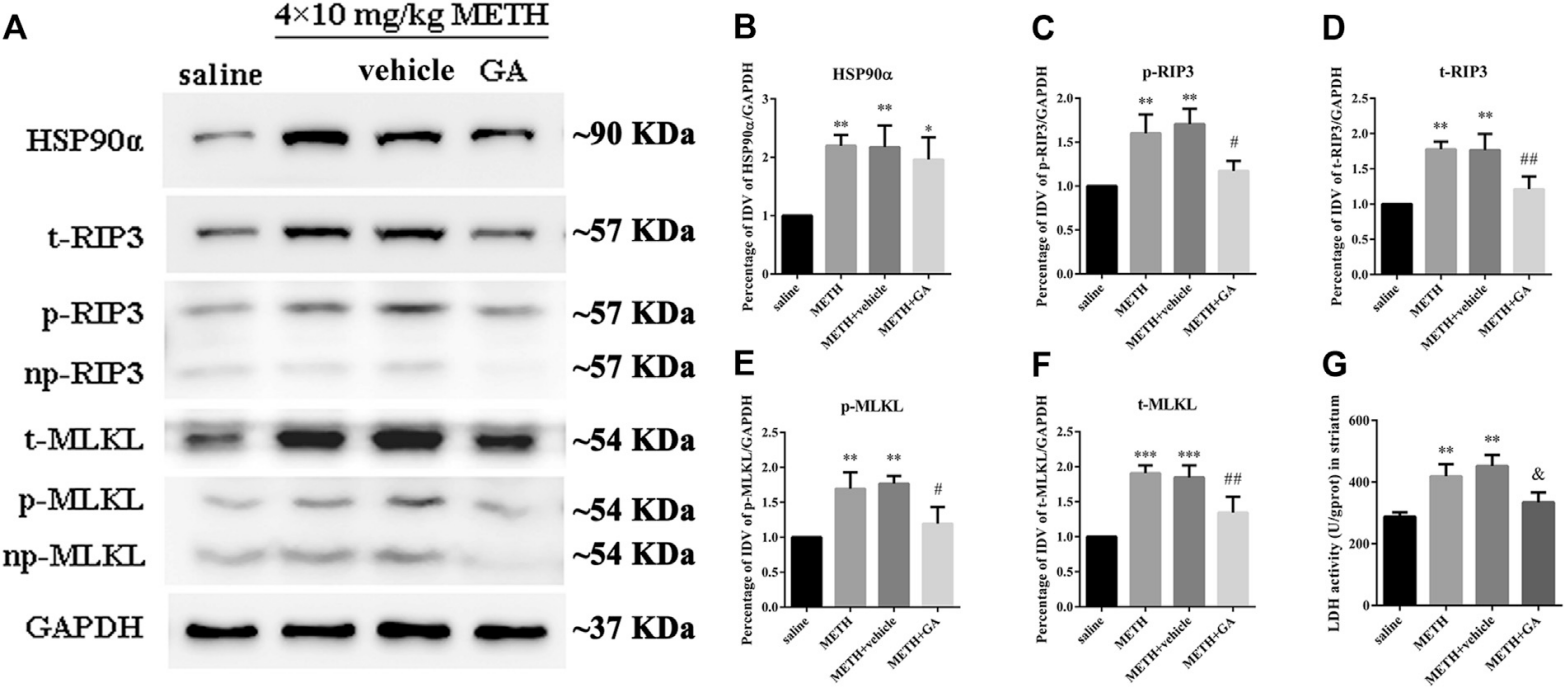
HSP90α inhibition by brain stereotactic injections of GA attenuated the METH/HT-induced upregulation of RIP3, p-RIP3, MLKL, and p-MLKL and the LDH activity in the rats’ striatum following a binge dose of METH. **(A)** WB of HSP90α, RIP3, p-RIP3, MLKL, and p-MLKL following a binge dose of METH administration and pre-treatment with GA by brain stereotactic injections. **(B–F)** Statistical analysis of WB of HSP90α, RIP3, p-RIP3, MLKL, and p-MLKL expression. Data were analyzed by one-way ANOVA, followed by a Tukey multiple comparisons posttest (*n* = 3). *p < 0.05, **p < 0.01, ***p < 0.001 vs. saline group; #*p* < 0.05, ##*p* < 0.01 vs. METH group. **(G)** Necrosis in the striatum of rats was determined by LDH cytotoxicity assay. Data were analyzed by one-way ANOVA, followed by a Tukey multiple comparisons posttest (*n* = 3). **p < 0.01 vs. saline group; &*p* < 0.05 vs. METH group.

To investigate whether the expression of HSP90α and RIP3 changed following METH and HT insults *in vivo*, we administered METH or a saline solution to rats and detected the expression of HSP90α and RIP3 1, 12, or 24 h after the last injection. The IF intensity of HSP90α (red) was increased 1 h after METH administration and was sustained even 24 h after the METH insult (Figure 8A). We did not detect obvious RIP3-positive (green) cells by immunostaining in the saline group but their number slightly increased after 1 h and significantly increased after 24 h in the METH group (Figure 8B). Generally, neuronal death is observed with one-day intervals in rats. After one day, cell death may no longer be evident as the dying cells may have undergone phagocytosis before lysis (Deng et al., 1999; Sabrini et al., 2019). Thus, we performed our experiments 24 h after the METH insult. The phos-tag SDS-PAGE results showed that p-RIP3, RIP3, p-MLKL, and MLKL in rat striatum cells were dramatically up-regulated 24 h after METH and METH + vehicle administration compared to that in the saline groups. On the other hand, HSP90α inhibition significantly blocked the expression of the METH/HT-induced canonical necroptotic molecules (Figures 9A–F). LDH cytotoxicity assays *in vivo* were also conducted. Compared with the saline group, we observed an increased LDH release in the METH and METH + vehicle groups. However, the increased LDH release was decreased in the GA pre-treatment groups (Figure 9G). These results demonstrate that HSP90α inhibition can partially protect striatal neurons from METH/HT-induced necroptosis.

## Discussion

In this study, we first demonstrated that METH and HT insults might lead to the upregulation of HSP90α, RIP3, and MLKL. Furthermore, the upregulation of HSP90α induced by METH and HT plays a regulatory role in the phosphorylation of RIP3 and subsequent necroptosis. Finally, by using an animal *in vivo* model, we demonstrated the role of HSP90α in METH/HT-induced necroptosis in the rat striatum. These results provided potential therapeutic targets and clinical diagnostic biomarkers for future use.

METH-induced HT allegedly results from the activation of dopaminergic (Mechan et al., 2002) and serotonergic (Herin et al., 2005) receptors in the thermoregulatory circuits of the hypothalamus, the direct or indirect activation of the sympathetic nervous system, the loss of vasoconstriction-mediated heat dissipation (Sprague et al., 2018), cerebrovascular damage, an increased level of oxidative stress and calcium entry, which contribute to METH-induced neurotoxicity in a dose-dependent manner (Miller and O’Callaghan, 2003; Yamamoto et al., 2010). Humans presented pathological HT during acute intoxication by METH (Kojima et al., 1984; Buffum and Shulgin, 2001; Marco et al., 2021). Additionally, HT can also markedly promote METH-induced neurotoxicity in rodents and non-human primates in similar ranges (Crean et al., 2006; Crean et al., 2007; Gutierrez et al., 2018). Thus, animal *in vivo* and *in vitro* models have been highly useful in identifying the neurochemical and physiological mechanisms of METH-induced HT. Several dose regimens of METH administration have been evaluated in rodent studies, i.e., single high dose (40 mg/kg) or binge doses (4 × 10 mg/kg, 2–3 h intervals), and escalating doses (1–10 mg/kg, twice a day, at 5 h intervals, for 10 days) of METH and chronic voluntary oral METH intake (Yang et al., 2018). The core body temperature of rats reached 38–39.5°C for 5 h after a single high dose of METH, while a binge dose (4 × 10 mg/kg, every 2 h, i.p.) caused an HT of 39–40°C for at least 6 h (Herring et al., 2008; Chauhan et al., 2014). In the present study, a binge dose of METH significantly increased the core body temperature to 38.5–39.5°C compared with the saline controls. The measured core body temperature was slightly lower than that observed in Chauhan et al.’s study, which may be caused by the differences in the experimental environment (Raineri et al., 2015), the weight of the rats (Bowyer et al., 1993), and the experimental equipment. Thus, we performed our HT experiments *in vitro* at 39.5°C.

HSP is a group of highly conserved proteins that respond to several stressors, including heat stress. They also play a role in cellular repair and the induction of thermotolerance (Yan et al., 2006). As an important chaperone molecule, HSP90 supports the folding of many important proteins, including signalling proteins and transcription factors. In response to stress, *HSP* gene expression is activated by *cis*-acting promoter elements which consist of variations of an inverted repeat sequence (nGAAn) called heat shock elements (HSE) and a homotrimeric DNA-binding transcription factor--heat shock factor 1 (HSF1) in eukaryotic cells (Ahn and Thiele, 2003). The denatured protein produced by heat shock or other types of stress, creates binding sites for HSP90 and changes the balance such as to release HSF1, then activated HSF1 binds to HSEs, HSEs are required to induce the expression of many genes central to the proteostasis network, including general chaperones of the heat shock protein classes HSP70 and HSP90 (Xiao and Lis, 1988; Lepock, 2005). In turn, cytosolic HSP70 and HSP90 have both been implicated in the negative regulation of HSF1 activity (Akerfelt et al., 2010). HSP90α is an isoform of HSP90, which plays an essential role in the response to external stimuli (Grad et al., 2010). For HSP90α gene expression, Zhang et al. reported that the 5’ flanking sequences play a critical role in both constitutive expression and stress-induced expression of the human HSP90α gene (Zhang et al., 1999). HSP90α expression and extracellular secretion increase rapidly to protect the cells from damage in response to elevated temperature, infection, or oxidative stress (ROS) (Chatterjee et al., 2007). However, some studies indicated that the up-regulation of HSP90α could stabilize death-related proteins that mediate cell death (Li et al., 2015; Li et al., 2016). In our study, the co-treatment with METH and HT increased HSP90α expression more than HT or METH alone. The inhibition of HSP90α partially protected the primary cultures of striatal neurons from METH/HT injuries. These results suggest the regulatory role of the high expression of HSP90α in promoting METH/HT-induced injuries in striatal neurons.

In this study and many others, the concentration of METH used to promote cell death is in the millimolar range, which is several orders of magnitude higher than that in the blood of abusers. For example, the mean blood concentration of METH in human abusers (e.g., arrested by police in Kern County, CA) was estimated at 2.0 μM (*n* = 105) with a maximum of 11.1 μM (Melega et al., 2007). Melega et al. also reported that the concentration of METH in the blood and brain necessary to induce neurotoxicity *in vivo* after intravenous administration (1–5 mg/kg) varies between 1 and 10 μM. However, METH is distributed preferentially in the brain rather than in the plasma. Thus, the concentration of METH in the brain should be higher than in the blood (Melega et al., 1995). The concentrations of METH in the frontal cortex, striatum, and cerebellum of rats were more than 10-fold higher than in the plasma (Melega et al., 1995). In humans, the common dose of a well-adopted abuser is 1 g or more METH per day (Simon et al., 2002; Melega et al., 2007). In these cases, the blood concentration of METH may increase to the millimolar range (Badisa et al., 2019). Meanwhile, systemic responses, such as immune responses and HT, might play crucial roles in METH-induced toxicity *in vivo* (Papageorgiou et al., 2019; Marco et al., 2021). Therefore, the concentration of METH used to promote direct neurotoxicity *in vitro* should be higher than the one required *in vivo*. A millimolar range concentration is often used to study the mechanism of METH neurotoxicity in culture studies (Huang et al., 2009; Chen et al., 2020). In our study, we administered 4 mM METH, which is similar to several studies investigating METH-induced neurotoxicity (Huang et al., 2015; Huang et al., 2017). Moreover, we observed a particularly obvious increase in necrotic cell death 6 h after treatment with 4 mM METH, suggesting a high level of neuronal cytotoxicity. Therefore, we used this concentration to mimic the impact of high doses of METH in individuals who are acutely exposed to the substance. The sub-toxic effects of lower doses of METH (0.1, 0.5, and 1 mM) on neurons will be investigated in our future research. In our *in vivo* experiments, we administered binge doses of METH because they can cause more severe damage to the neurons compared to a single METH administration (Ares-Santos et al., 2014). This mode of administration is also closer to an overdose in humans (Davidson et al., 2001).

METH primarily affects multiple functional areas in the human brain (Lu et al., 2019). The striatum is associated with movement disorders and is involved in the control of attention, executive function, motivated behaviours, and neuropsychiatric conditions, such as compulsive disorders, psychoses, and addictive behaviours (Zhu et al., 2006; Granado et al., 2013; Potvin et al., 2018). METH exposure can cause neuronal apoptosis and autophagy. A loss of approximately 25% of striatal neurons has been reported 24 h after METH exposure (Zhu et al., 2005). Many studies indicated that the death of striatal neurons occurred by apoptosis and autophagy after METH exposure (Zhu et al., 2006). For example, C/EBPβ was involved in METH-induced DDIT4-mediated neuronal autophagy and Trib3-mediated neuronal apoptosis (Huang et al., 2019). Xu et al*.* suggested that nuclear protein 1 (Nupr1/com1/p8) was involved in neuronal apoptosis and autophagy caused by high doses of METH through the endoplasmic reticulum (ER) stress signalling pathway (Xu et al., 2017). However, the inhibition of these molecules cannot protect all the neurons, indicating that apoptosis and autophagy may mediate the degeneration of only some of them. Since necrosis was discovered, it was mainly believed that it was a form of cell death that cannot be accurately intervened. When the necroptosis process was discovered, the research on necrosis received more attention. Multiple molecules are involved in necroptosis. The TNFα-regulated pathway, which is mediated by RIP1, RIP3, and MLKL is the most extensive and important (Sun et al., 2012; Liao et al., 2017; Ruan et al., 2019; Wang et al., 2020; Wu et al., 2020). This pathway is briefly described as follows: the death ligands bind to the corresponding receptors to pass the death signal into the cells. RIP1 can then bind RIP3 in the cytoplasm to form complex-II, which in turn promotes the phosphorylation of RIP3. This may cause an excessive accumulation of ROS (Chtourou et al., 2015) and the aggregation and translocation of phosphorylated MLKL to the cell membrane to form pores. The formation of these pores can deregulate the balance in the concentration of metal ions inside and outside the cell membrane and eventually promote cell necrosis (Cho et al., 2009; Sun et al., 2012). Our previous study showed that treatment with 4 mM METH for 12 h induced necroptosis in the cortical neurons of rats *in vitro* (Xiong et al., 2016), and cortical neurons showed signs of necroptosis after treatment with 1 mM METH at 39°C (Guo et al., 2020). Additionally, Zhao et al. reported that necroptosis occurred in the striatum of human and mice brain samples exposed to METH and the RIP3/MLKL/Drp1 pathway played an essential role in the mechanism of METH-induced neuronal programmed necrosis (Zhao et al., 2021). However, it is still unclear whether METH-induced HT can induce necroptosis in striatal neurons. In this study, METH combined with HT triggered necroptosis in striatal neurons after 6 h. The inhibition of HSP90α decreased the METH/HT-induced upregulation of p-RIP3, RIP3, p-MLKL, and MLKL, suggesting that HSP90α may mediate necroptosis by regulating the phosphorylation of RIP3. Interestingly, pyroptosis, an inflammasome-associated regulatory necrosis, is closely associated with the pathogenesis of neurodegenerative diseases (Wang et al., 2019; Huang et al., 2021) and METH induces ER stress that mediates GSDME-dependent pyroptosis in hippocampal neuronal cells (Liu et al., 2020). That is to say, METH abuse may cause a variety of regulatory cell necrosis. We postulate that different regulatory necrosis can be triggered under METH/HT injuries and the neural cells can experience extensive crosstalk between different types of cell death. Further research is needed to clarify this hypothesis.

In conclusion, our results indicated that HSP90α had a significant impact on METH/HT-induced necroptosis in striatal neurons. These results provide a deeper understanding of the regulatory mechanism of METH/HT-induced injury.

## Data Availability

The original contributions presented in the study are included in the article/Supplementary Material, further inquiries can be directed to the corresponding authors.
