# Supplementary material for: The Role of HSP90α in Methamphetamine/Hyperthermia-Induced Necroptosis in Rat Striatal Neurons
Source: Front Pharmacol. 2021 Jul 19;12:716394. doi: 10.3389/fphar.2021.716394 (PMC8326403; doi:10.3389/fphar.2021.716394)
Supplement: Supplementary file 1 [file DataSheet1.pdf]

Supplementary Material

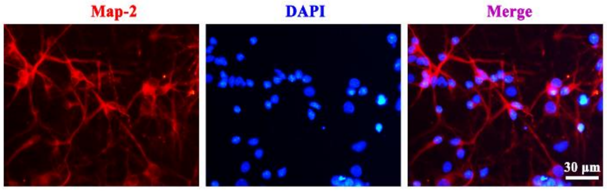

Supplementary Figure 1. Detection of Map-2-positive cells in the primary cultures of striatal neurons on the 7th day of *in vitro* culture. The red fluorescence represents Map-2 protein expression, DAPI (blue) was used for nuclear staining. *Scale bar*=30 μm.

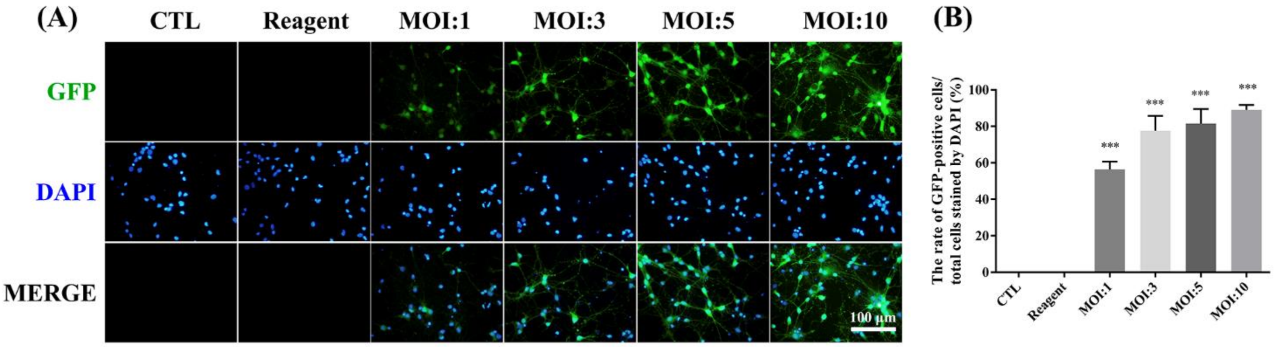

Supplementary Figure 2. The appropriate lentivirus concentration used in the primary cultures of striatal neurons. **(A)** Primary cultures of striatal neurons infected with the negative control lentivirus (hU6-MCS-Ubiquitin-EFGP-IRES-puromycin) at MOI:1, MOI:3, MOI:5, and MOI:10 for 72 h. The green fluorescence represents GFP protein expression, DAPI (blue) was used for nuclear staining. *Scale bar*=100 μm. **(B)** The lentivirus infection rate in striatal neurons. Data were analyzed by one-way ANOVA, followed by a Tukey multiple comparisons posttest (n=3). *** $p < 0.001$ *vs.* CTL group.
